# Evolutionary Determinants of Genetic Variation in Susceptibility to Infectious Diseases in Humans

**DOI:** 10.1371/journal.pone.0029089

**Published:** 2012-01-05

**Authors:** Christi Baker, Janis Antonovics

**Affiliations:** Department of Biology, University of Virginia, Charlottesville, VA 22904, USA; Biomedical Research Institute, United States of America

## Abstract

Although genetic variation among humans in their susceptibility to infectious diseases has long been appreciated, little focus has been devoted to identifying patterns in levels of variation in susceptibility to different diseases. Levels of genetic variation in susceptibility associated with 40 human infectious diseases were assessed by a survey of studies on both pedigree-based quantitative variation, as well as studies on different classes of marker alleles. These estimates were correlated with pathogen traits, epidemiological characteristics, and effectiveness of the human immune response. The strongest predictors of levels of genetic variation in susceptibility were disease characteristics negatively associated with immune effectiveness. High levels of genetic variation were associated with diseases with long infectious periods and for which vaccine development attempts have been unsuccessful. These findings are consistent with predictions based on theoretical models incorporating fitness costs associated with the different types of resistance mechanisms. An appreciation of these observed patterns will be a valuable tool in directing future research given that genetic variation in disease susceptibility has large implications for vaccine development and epidemiology.

## Introduction

‘It seems likely that when a species is subjected to a series of attacks by an evolving parasite it may be forced along a path of structural change by its temporarily successful acquisitions of immunity. But in the end it may be driven, so to say, into a corner, where further immunity involves structural changes which are disastrous to it in its everyday life.’

J.B.S. Haldane (1932) *The Causes of Evolution*.

In the middle of the last century, Haldane made his now-famous assertion that infectious disease acts as a selective force in human populations, suggesting that heterozygosity for certain blood disorders might confer a selective advantage in the form of resistance to malaria infection [1]–[3]. Empirical evidence in support of this prediction soon followed [4]–[7], even though this assertion was initially met with strong opposition within the scientific community (J. J. Murray, personal communication). Since the post-genomic era, research programs have been built around the search for genetic diversity associated with infectious disease, the findings of which have been documented in many reviews and texts [8]–[16]. Highlighting the importance of a genetic component to disease susceptibility, Sorensen et al. (1988) showed that adult adoptees were five times more likely to die from an infectious disease if their biological parent also died from an infection; this increased risk associated with infectious disease was higher than that for either cardiovascular disease or cancer [17].

Infectious disease remains a leading cause of death in many parts of the world, and efforts to identify genetic variation in disease susceptibility have been motivated by the implications for disease control strategies. Discoveries of genetic variation open up possibilities of pinpointing resistance pathways for targeted therapies and for personalized pharmaceuticals. Strong genetic components associated with susceptibility to natural infections may also be responsible for differences in vaccine response [18], [19], and point to the potential benefits of a more personalized vaccine approach [20], [21]. Genetic variation in susceptibility is also likely important for zoonotic diseases, a fact that was underscored by reports of H5N1 influenza infections clustering in family units, launching concerns that groups of susceptible individuals might provide zoonotic pathogens with a gateway into human populations [22], [23].

In contrast to the high levels of genetic variation in susceptibility to some infections, other well-characterized diseases, such as mumps, appear to be associated with very little variation in susceptibility [24], [25]. Nevertheless, there has been little attempt to develop a theoretical framework for understanding and predicting patterns in the levels of genetic variation for susceptibility to different diseases. For example, diseases with strong effects on host fitness, high prevalence levels, large geographic ranges, or long histories in human populations may generate high levels of transient genetic variation in susceptibility, but they may also lead to the loss of susceptibility genes over time and therefore eventual loss of variation depending on the costs. Alternatively, genetic variation in susceptibility may be the result of selection for multiple resistance mechanisms generated by correspondingly variable pathogen genotypes, as occurs in many gene-for-gene systems in plants. Furthermore, heterozygote advantage or pleiotropic effects with alternate resistance mechanisms could contribute to genetic variation in susceptibility. More recently, theoretical studies have proposed that the shape of the resistance-fitness trade-off curve is an important determinant of genetic diversity [26], but there has been no explicit test of this hypothesis.

**Figure 1:**
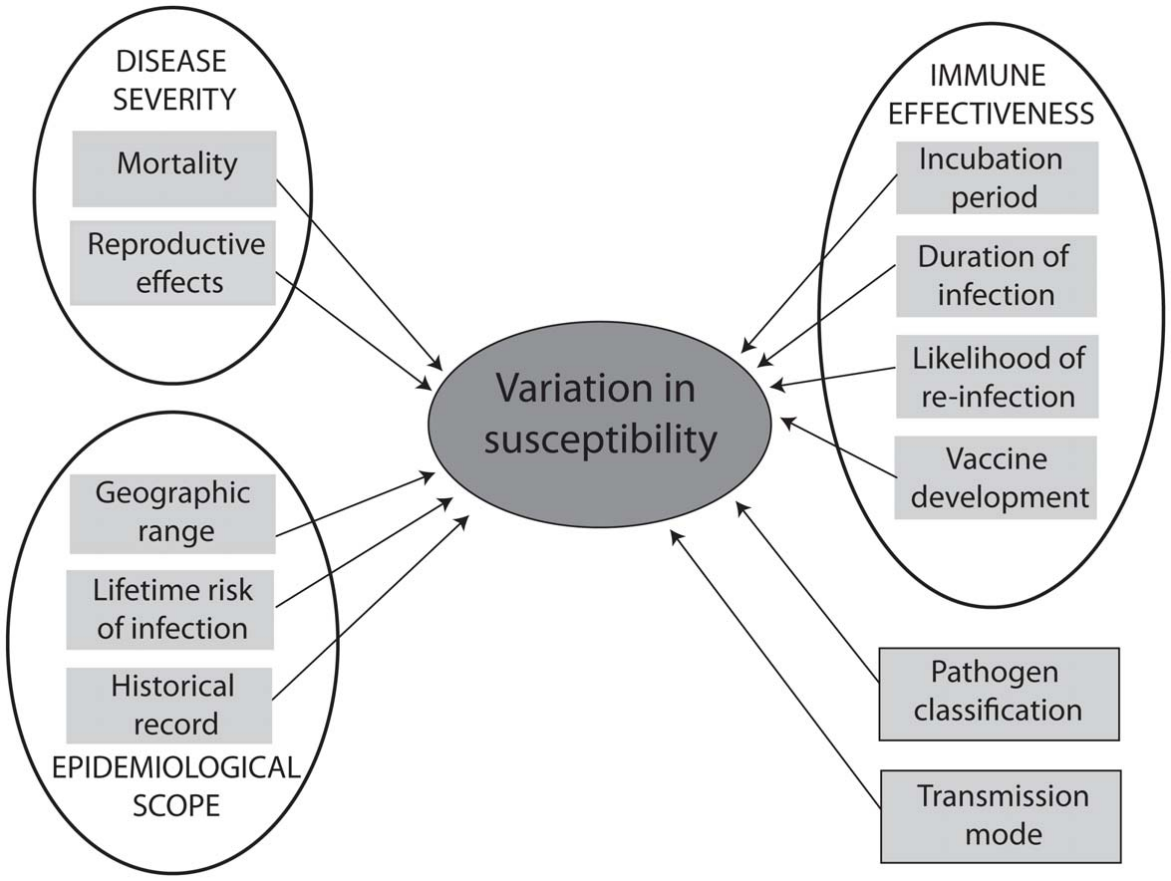
Diagram showing major disease characteristics and their component traits that were investigated in relation to genetic variation in infectious disease susceptibility.

In the present study, we estimate the relative magnitude of genetic variation in susceptibility to human diseases and relate this to disease characteristics in order to investigate the possible evolutionary forces responsible for the observed patterns in genetic diversity. We consider alternative hypotheses by focusing on measures of traits related to disease severity, epidemiological scope, and immune system effectiveness (Fig. 1). This study represents the first comparative analysis of a broad range of human pathogens seeking to identify evolutionary determinants of genetic variation in disease susceptibility.

## Results

We identified 46 diseases that met all of the criteria for inclusion in the study (see Methods). A total of 40 diseases were included in the final analyses because for six diseases, we could not find any scientific literature evaluating genetic variation in susceptibility (Table 1). The diseases included in the analysis were caused by diverse human pathogens (35% viruses, 32% bacteria, 12% protozoa, 5% fungi, and 15% helminths) with diverse transmission modes (10% sexual contact, 47% close contact, 30% indirect contact, and 12% vectors).

**Table 1:** List of diseases and corresponding pathogen species included in the analysis.

Disease	Pathogen	Acronym
African sleeping sickness	*Trypanosoma brucei gambiense*	ASS
Amebiasis	*Entamoeba histolytica*	AME
Ascariasis	*Ascaris lumbricoides*	ASC
Candidiasis	*Candida albicans*	CAN
Chickenpox	*Varicella zoster virus*	CHI
Cholera	*Vibrio cholerae*	CHO
Cryptosporidiosis	*Cryptosporidium parvum*	CRY
Dengue Fever	*Dengue virus*	DEN
Diphtheria	*Corynebacterium diphtheriae*	DIP
Elephantiasis	*Wuchereria bancrofti*	ELE
Gastric ulcers	*Helicobacter pylori*	ULC
Giardiasis	*Giardia lamblia*	GIA
Gonorrhea	*Neisseria gonorrhoeae*	GON
Hepatitis A	*Hepatitis A virus*	HPA
Hepatitis B	*Hepatitis B virus*	HPB
Hepatitis C	*Hepatitis C virus*	HPC
Herpes simplex 1	*Herpes simplex virus-1*	HS1
Herpes simplex 2	*Herpes simplex virus-2*	HS2
Hookworm	*Necator americanus*	HOO
Influenza	*Influenza A virus*	FLU
Leprosy	*Mycobacterium leprae*	LEP
Malaria	*Plasmodium falciparum*	MAL
Measles	*Measles virus*	MEA
Meningitis	*Haemophilus influenzae*	HIB
Meningitis	*Neisseria meningitidis*	NMG
Mononucleosis	*Epstein-Barr virus*	MON
Mumps	*Mumps virus*	MUM
Pertussis	*Bordetella pertussis*	PER
Pneumonia	*Streptococcus pneumoniae*	STP
Poliomyelitis	*Poliovirus*	POL
Ringworm	*Trichophyton rubrum*	RIN
Riverblindness	*Onchocerca volvulus*	RIV
Rubella	*Rubella virus*	RUB
Schistosomiasis	*Schistosoma mansoni*	SCH
Smallpox	*Variola virus*	SMA
Strep throat	*Streptococcus pyogenes*	STT
Trachoma	*Chlamydia trachomatis*	CHL
Trichuriasis	*Trichuris trichiura*	TRI
Tuberculosis	*Mycobacterium tuberculosis*	TUB
Typhoid fever	*Salmonella typhi*	TYP

Acronyms for each disease are as used in the figures.

### Univariate analyses of disease characteristics

**Figure 2:**
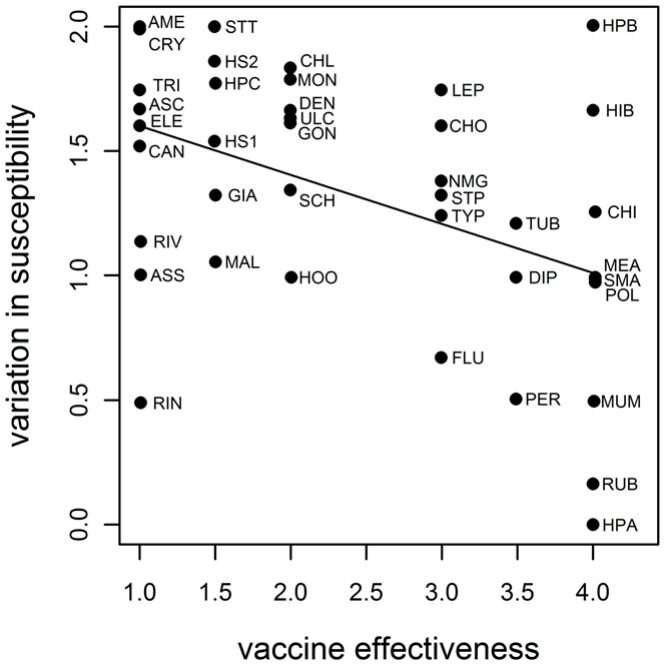
Relationship between levels of variation in susceptibility and effectiveness of vaccine development, as rank scores (*p* = 0.008). See Table 1 for key to acronyms for the individual diseases.

We first conducted univariate analyses to investigate the effects of the individual disease characteristics on variation in susceptibility. Variation in susceptibility was significantly negatively correlated with effectiveness of vaccine development (*r_s_* = −0.41, *p* = 0.008; Fig. 2), with low levels of genetic variation for diseases with an effective vaccine. Amebiasis, trichuriasis, ascariasis, and strep throat were among the diseases with high levels of variation in susceptibility for which no vaccines have been successfully developed. Diseases successfully combated by vaccines, such as hepatitis A, rubella, mumps, and pertussis, exhibited low levels of variation in susceptibility.

**Figure 3:**
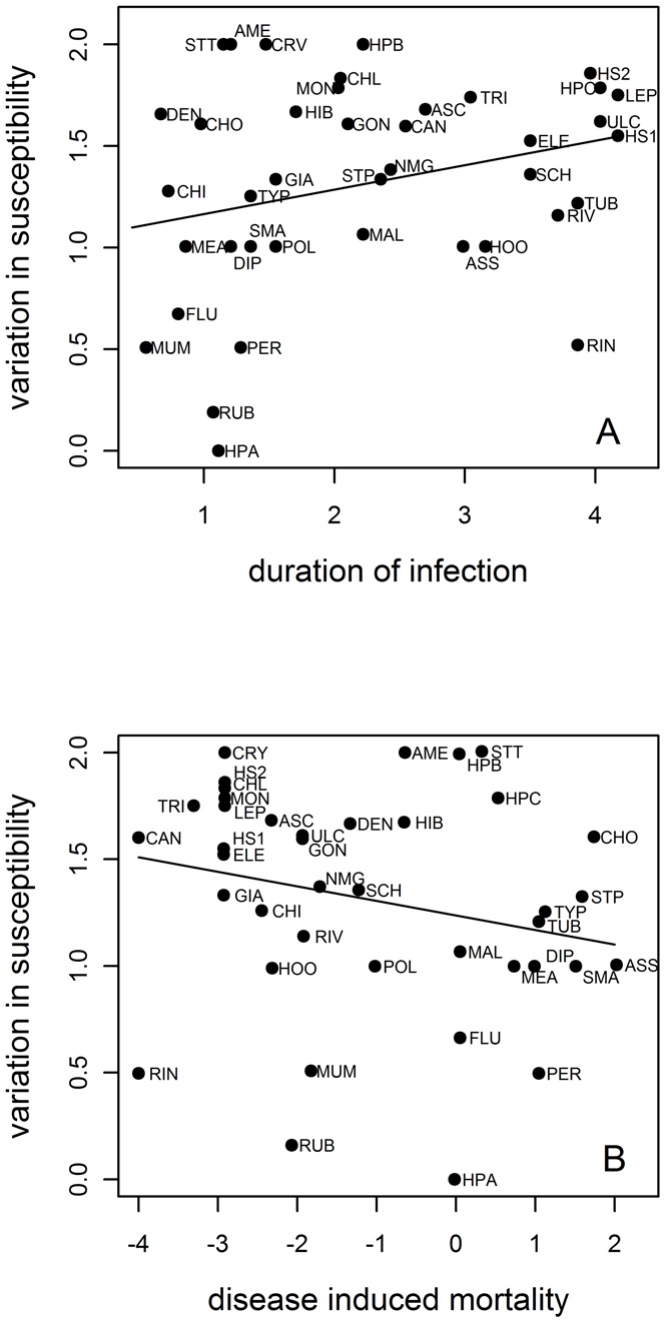
Relationship between levels of variation in susceptibility and disease traits for 40 human diseases. (A) Duration of infectious period, as log days (*p* = 0.09). (B) Disease-induced mortality, as log case fatality rate (*p* = 0.07). See Table 1 for key to acronyms for the individual diseases.

Marginally significant correlations were observed in susceptibility with duration of the infectious period (*r_s_* = 0.26, *p* = 0.09; Fig. 3A) and with mortality (*r_s_* = −0.29, *p* = 0.07; Fig. 3B). Diseases exhibiting high levels of genetic variation in susceptibility were generally those with long infectious periods, such as herpes simplex viruses (HSV-1 and HSV-2), leprosy, hepatitis C, and gastric ulcers. Diseases with the lowest levels of variation were of short duration, and included hepatitis A, rubella, mumps, influenza, and pertussis. Still, some diseases with short infectious periods showed evidence for high levels of genetic variation, including cholera and dengue fever. Diseases that cause high mortality, such as African sleeping sickness, cholera, streptococcal pneumonia, and smallpox were associated with lower levels of variation in susceptibility than were more benign infections, such as candidiasis, trichuriasis, mononucleosis and elephantiasis.

**Figure 4:**
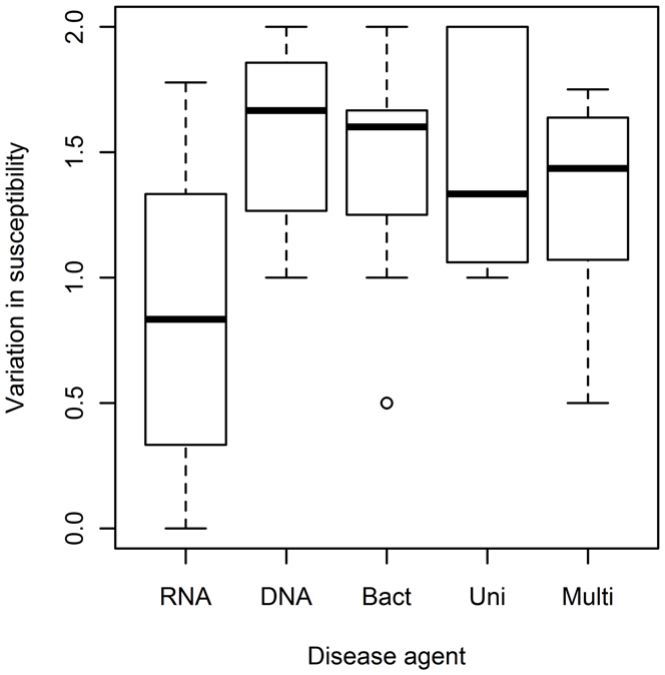
Levels of variation in susceptibility among different classes of disease agents (*F*_4,35_ = 2.82, *p* = 0.04). DNA = DNA viruses, RNA = RNA viruses, Bact = bacteria, Uni = unicellular, i.e. protozoans. Multi = multicellular, i.e. fungi and helminths. Boxes depict inter-quartile ranges (IQR), and bold horizontal lines indicate medians for each group. Whiskers extend from the minimum to maximum value. Circle represents an outlier, defined as a value smaller than 1.5 times the IQR from the first quartile.

A one-way ANOVA revealed that levels of variation in susceptibility were significantly different among the classes of disease agents (*F*_4,35_ = 2.82, *p* = 0.04, Fig. 4). There tended to be lower levels of variation in susceptibility to diseases caused by RNA viruses than to diseases caused by DNA viruses and bacteria, though the pairwise effects between them were not significant (at the α = 0.05 level in a Tukey HSD test correcting for multiple post hoc comparisons).

Levels of variation in susceptibility were not significantly different among the disease transmission modes (*F*_3,36_ = 1.59, *p* = 0.21).

### Multivariate analysis of disease characteristics

Because the three major traits of interest namely disease severity, epidemiological scope and immune effectiveness (Fig. 1) were each measured by several inter-correlated explanatory traits, we used principal components analysis (PCA) to identify combinations of these traits (represented by principal component scores) that contributed independently to each variable. We then tested whether the first two PCA scores for each of these major traits explained genetic variation in susceptibility. This was done using an ANCOVA, with disease agent and transmission mode as class variables, and a backward selection procedure that sequentially removed terms contributing least to the variance in susceptibility. This analysis only retained immune effectiveness and disease agent class as factors contributing to genetic variation in susceptibility (*p*<0296, and *p*<0.0150 respectively, Type III sums of squares).

**Figure 5:**
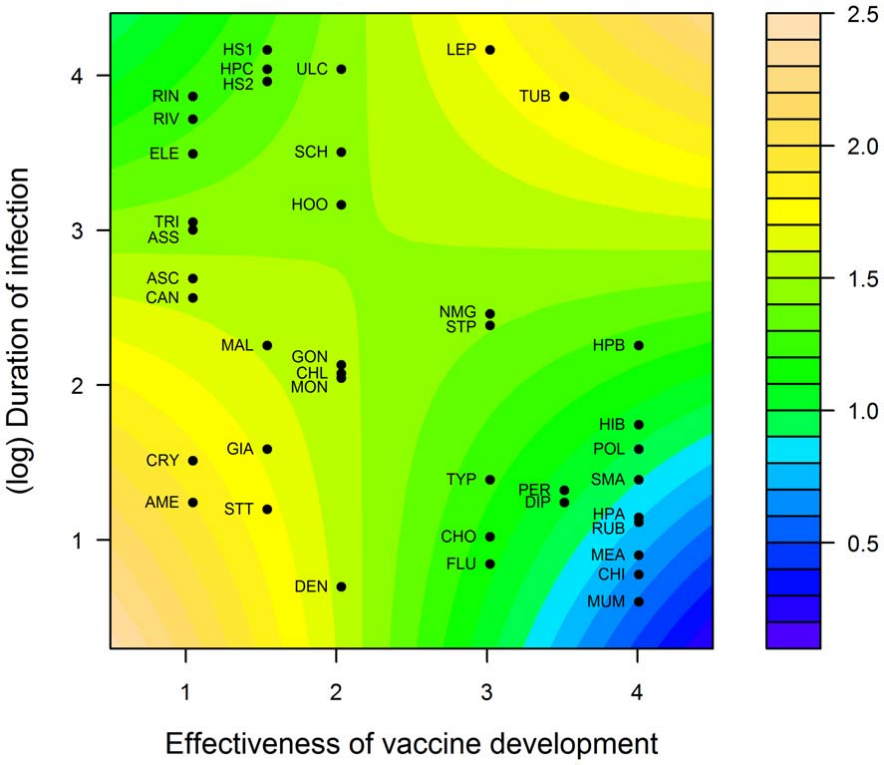
Levels of genetic variation in susceptibility in relation to the effectiveness of vaccine development and the duration of the infectious period. Color key indicates level of variation in susceptibility. Purple/blue corresponds to low levels of variation in susceptibility, while yellow/orange corresponds to high levels of variation in susceptibility. Response surface is based on a multiple regression model (see text for explanation).

As only immune effectiveness was significant, we performed a multiple regression of level of variation in susceptibility on the individual immune system traits. This analysis indicated that the duration of the infectious period and effectiveness of vaccine development, as well as an interaction between these terms, were highly significant predictors of variation in susceptibility (Table 2). A plot of the interaction between the duration of the infectious period and the effectiveness of vaccine development showed that high levels of genetic variation in susceptibility were associated with diseases of longer duration, particularly for diseases with effective vaccine development (Fig. 5). Very low levels of genetic variation in susceptibility were associated with diseases with short infectious periods and for which effective vaccines have been developed. However, where vaccine development had been particularly unsuccessful, even diseases of short duration often showed substantial variation in susceptibility.

**Table 2:** Analysis of covariance on levels of variation in susceptibility.

Source	d.f.	Type III SS	*F*	*p*
Agent	4	1.46	3.13	0.029
Transmission	3	0.52	1.51	0.234
Duration	1	1.23	10.61	0.002
Vaccine	1	2.80	24.00	<.0001
Duration*Vaccine	1	1.25	10.77	0.002
Error	29	3.38		
Total	39	10.22		

Agent = disease agent (as a class variable), Transmission = transmission mode (as a class variable), Duration = duration of the infectious period, Vaccine = effectiveness of vaccine development.

## Discussion

The present study indicates that there are large differences among infectious diseases in the levels of genetic variation for susceptibility to those diseases. The differences we found, at least with regard to *relative* levels of variation, were consistent with previous estimates carried out by independent observers, and with the coverage attributed to given diseases in textbooks reviewing evidence for genetic variation (see Methods section on Validation). Comparisons of our estimates with these independent assessments suggest consensus on high levels of genetic variation in susceptibility to diseases such as leprosy, mononucleosis, and gastric ulcers, intermediate levels with diseases including polio, chickenpox, and smallpox, and low levels of variation in susceptibility to hepatitis A, mumps, and pertussis.

The levels of variation in susceptibility were strongly associated with particular disease traits. The disease characteristics that consistently predicted levels of variation in susceptibility were those that reflected the effectiveness of the immune response to disease. This was true for all diseases and independent of the type of pathogen, its transmission mode, or levels of disease severity. As with the scores for levels of variation, we validated our estimates of disease characteristics with independent evaluations. Thus, there was strong agreement between our estimates and those collected independently by an upper-level class for all of the comparable disease traits (unpublished data). Therefore whereas caution is needed in interpreting the data for any specific disease (and these interpretations may be approximate), the consistency of the estimates across several studies indicates that the overall patterns found in this study are robust.

While our analyses indicate that characteristics related to the effectiveness of the adaptive immune response are important determinants of levels of variation in susceptibility, the traits related to the immune response were not equally powerful predictors. The finding that the relative success of vaccine development was the trait most strongly correlated with levels of variation in susceptibility seems curious, as successful vaccine development cannot be, in and of itself, an evolutionary determinant; the introduction of vaccination regimes is too recent to have substantially influenced extant patterns of human genetic variation. Rather, it is likely that estimates for the trait ‘vaccine development’ best reflect the effectiveness of an inducible immune response to a given infectious disease organism, and that vaccines are most easily developed against a disease for which there is also a natural long-lasting immune response.

The observed patterns can be interpreted in the light of several hypotheses that are not necessarily exclusive, nor even the only possibilities. We consider these in turn.

### Fitness effects

It could be argued that diseases with strong fitness effects on infected individuals (high mortality and sterility) and with a large epidemiological impact (high lifetime risk of infection, large geographic range, and long historical record of disease in human populations) should be associated with high levels of genetic variation. However, our data do not support this hypothesis, as characteristics estimating disease severity and epidemiological scope were not strong predictors of levels of genetic variation. While severe and/or widespread diseases might generate genetic variation by producing strong selection for resistance alleles, diseases with extreme fitness effects may ultimately eliminate variation by fixing resistance alleles. Therefore, the relationship between disease severity and epidemiological traits and resulting levels of genetic variation in susceptibility may be non-linear, but we also found no evidence of this. There was no trend in the data suggesting that diseases of intermediate severity (representing perhaps transient polymorphisms in resistance) had the greatest amount of genetic variation.

### Variation in pathogen genotypes

The most polymorphic gene complex in the vertebrate genome is the major histocompatibility complex (MHC), and population geneticists have debated at length the forces that maintain variation in these immune-related genes [27], [28]. While it is generally agreed that this diversity is likely to be maintained by pathogen-mediated selection, the specific mechanisms (e.g. overdominance, rare-allele advantage, and fluctuating selection) often generate similar predictions that are difficult to distinguish, so there is no consensus about which mechanisms are the most important or the relevance of pathogen genotypic variation [29], [30]. Unfortunately, it was not possible to compare the levels of genetic diversity in the pathogens causing the different diseases in this study for three reasons. First, although there is abundant evidence for general variation in these pathogens (in the form of, for example, strain differences or antigenic variants), it was usually unclear how meaningful this variation was for selection on human genotypes. Second, it was difficult to obtain valid comparative measures of strain differences across pathogen taxa as different as, say, viruses and helminths. Third, any impact of pathogen variation on host genetic variation requires the presence of host genotype×pathogen genotype interaction in infectivity or disease expression. Evidence of such interactions (analogous to the gene-for-gene systems seen in crop plants) would normally require experimental cross-inoculation experiments, and are therefore difficult to obtain except where there are clear and well-understood observable differences. One example, albeit at the species level, is the lower susceptibility of Duffy blood groups to malaria caused by *Plasmodium vivax*, yet continued susceptibility to *Plasmodium falciparum*.

### Resistance costs

Mathematical models of host-parasite co-evolution show that that genetic variation in the host can be maintained in the absence of pathogen variation and over a wide range of costs (manifested in the absence of disease as lower fitness of resistant individuals compared to susceptibles), particularly when alleles confer extreme phenotypes of susceptibility in the population [31], [32]. Costs of resistance mechanisms have often been assumed, as it seems intuitive that the energetic expense of mounting such resistance must detract from a finite pool of resources that could otherwise be employed for survival or reproduction. Resistance costs have been demonstrated in a number of experimental systems [33]–[37], but only rarely in nature [38]. Fitness costs to genes that result in various forms of thalassemia and provide resistance to malaria are also well known [39], [40].

More recent studies have expanded single-locus models of costs to consider the more likely scenario in which relative susceptibility to disease is a complex quantitative trait determined by allelic variation at multiple loci. The resulting selective forces depend heavily on the shape of the resistance-fitness trade-off curve, and this shape is a crucial evolutionary determinant of the resulting level of genetic variation in resistance [26], [41]. Costs can be accelerating, such that increases in resistance become increasingly costly, or decelerating, where accumulating resistance becomes decreasingly costly. When the trade-off curve is accelerating, there will be a stabilizing component in the resulting selection gradient that will reduce genetic variation. Alternatively, a decelerating trade-off will result in disruptive selection and increased genetic diversity.

Although the trade-off curves have not been characterized for any human diseases (see [38] for an experimental study with a plant pathogen), the shapes of these curves can be inferred from our understanding of the mechanisms conferring resistance. Specifically, mechanisms associated with the adaptive immune system should have accelerating costs, as the basic immune system costs are generally similar and low among individuals in the absence of disease, and therefore further genetic gains in resistance may require costly structural changes. Alternatively, where the inducible immune response is ineffective, resistance mechanisms may require constitutive mutations, which are likely to be costly. Based on these inferences, we can predict that where the adaptive immune system is effective, there will be accelerating resistance-fitness trade-offs which will generate stabilizing selection and low levels of genetic variation. On the other hand, the decelerating trade-off associated with ineffective adaptive immune responses should lead to destabilizing selection and high levels of genetic variation.

Our findings are consistent with these expectations. Diseases that evoke effective adaptive immune responses, as evidenced by traits such as successful vaccine development and short infectious periods, result in low levels of variation in susceptibility in their hosts. Likewise, diseases that circumvent the adaptive immune system, defying attempts at vaccine development and exhibiting long infectious periods, should show decelerating costs, and indeed are associated with high levels variation in susceptibility. As resistance-fitness trade-off curves have not been characterized for any human diseases to date, we cannot definitively link the differences in levels of genetic variation with differences in the trade-off curves; however, there is precedence in the literature for using observed patterns of genetic variation in susceptibility to imply the shapes of these trade-offs [42]. Measuring such trade-off curves for human diseases has hardly been contemplated, but presents an important research challenge.

Our study also provided important methodological lessons (see Information S1 for further discussion). It was clear that quantifying levels of genetic variation in disease susceptibility in human populations requires a comprehensive approach. Currently, the majority of studies on variation in susceptibility are case-control association studies that report the magnitudes of allelic effects, with the primary goal of pinpointing important loci involved in pathogenesis. For estimation of how much these loci contribute to overall genetic variation, information is additionally required on the allele frequencies of the loci as well as the risk of exposure to the disease in the population being investigated. Usually, such information is not reported. If non-candidate gene markers such as SNPs are being used, estimates of the locus-specific contribution to overall variation in susceptibility raise further methodological issues that have been discussed under the rubric of so-called “missing heritability” [43]. Databases of SNP data and of data pertaining to specific diseases are beginning to emerge, but we know of no single database that includes both pedigree-based and marker-based data for a large array of diseases.

Perhaps most surprising, was the difficulty of obtaining good quantitative data on many of the disease characteristics (see Information S1, for further discussion). This difficulty has also been mentioned in previous comparative studies of human sexually transmitted infectious diseases [44]. One would imagine that the characteristics of different human diseases would be well understood, and that there would be ‘canonical’ information on these diseases in readily available databases. Some steps have been made towards this in recent databases such as GIDEON, where comprehensive information is available especially on geographical distribution and prevalence; this has been invaluable in comparative studies of human disease incidence and genetic variation [45], [46]. However, most of the databases (including GIDEON) understandably have a focus on both diagnosis and treatment. Frequently we could not find clear data (both means and variances) for basic disease characters; for example, mortality rate was generally only reported as rates for hospitalized patients. For other traits, such as disease duration and effects on fertility, there was simply very little quantitative data available.

Our results also have implications for prioritizing research on genetic variation in response to human diseases. Malaria and tuberculosis have been the major focus of efforts to identify genetic variation in disease susceptibility, and these two diseases have commanded the lion's share of attention in this respect. In stark contrast, a number of diseases we would predict to be associated with high levels of variation in resistance based on their long infectious periods and failed vaccine development efforts are under-represented among studies of genetic diversity, even though there is evidence to suggest that this predicted variation exists. It is not surprising that many of these diseases—African sleeping sickness, dengue fever, trichuriasis and hookworm—are classified as “neglected tropical diseases” by the WHO and are diseases associated with extreme poverty. As renewed efforts to eradicate these neglected diseases have been invigorated by recent high-profile donations by The Gates Foundation and others, research on genetic variation in susceptibility may help to identify novel strategies for controlling these infections.

## Methods

### Ethics statement

The research did not involve human or animal participants.

### Selection of diseases

Diseases included in the study are shown in Table 1. Analyses only included diseases with long-standing evolutionary relationships with human hosts and did not include diseases that emerged during the last century. Diseases that were not primarily human-to-human transmitted were also excluded to minimize the confounding effects of pathogen evolution in response to an alternate host. For diseases that are caused by multiple etiological agents, a single causative pathogen species was selected to be the focus of study. Analysis of a subset of infectious diseases suggested that only those with >2,000 citations in PubMed (as of 2010) were likely to have been studied with respect to variation in susceptibility; therefore, we set 2,000 citations as a threshold for identifying diseases to include in the study. Syphilis, typhus, yellow fever, rotavirus and shigellosis were also excluded because data on genetic variation was insufficient. The above guidelines for disease selection necessitated the omission of many important diseases. For example, leishmaniasis infects over 10 million people worldwide but was excluded because the majority of human disease is caused by transmission from other animals and because of the confounding effects of multiple etiological agents that are not always clearly identified in published research. Similarly, although HIV causes approximately 2 million deaths each year, AIDS was not included because of its relatively recent emergence in human populations.

### Scoring genetic variation in disease susceptibility

We collected data related to genetic variation in disease susceptibility by conducting web-based searches of several databases including PubMed and Web of Science. We used search terms related to genetic variation (“polymorphism”, “genetic resistance/susceptibility”) or the type of study (“twin study”, “case control study”) in combination with disease and pathogen names. We traced the references in these papers to identify relevant prior studies, those published in books, and also identified subsequent work using citation indices.

In assessing levels of genetic variation, we aimed to directly or indirectly estimate the proportion of observable variation in disease susceptibility that is due to additive genetic effects, i.e. the heritability [47]. We focused on evidence for genetic variation in infection rates rather than associations with disease severity or particular symptoms contingent on disease progression. Variation in disease expression may be due to host tolerance and recovery, and the genetic determinants of these may or may not be related to the genetic determinants responsible for susceptibility to infection *per se*.

Because our interest was in evolutionary processes, we did not consider genetic variation to human disease in animal model systems, as they do not offer insight into evolutionary causes of variation in human populations. Data came from the following types of studies.

- Pedigree-based studies. These included investigations of disease concordance rates among monozygotic versus dizygotic twins, variance components analysis based on extended pedigrees, and analyses of among-population genetic variation in susceptibility. There are many caveats and cautions related to interpreting these studies, and the major ones are summarized in Information S1.
- Marker-based studies. Evidence for variation in disease-susceptibility loci was mostly obtained from case-control association studies and genome-wide linkage studies. Four classes of genetic marker were distinguished among the marker-based studies: (a) human leukocyte antigen (HLA) markers, (b) non-HLA immune markers, (c) neutral marker associations (including single nucleotide polymorphism (SNP) associations and linkage studies), and (d) directed candidate gene markers for which there is evidence of a functional role in disease susceptibility.

Estimates for marker-based studies were based on a weighted mean (see below for description of weights) of the scores assigned to each type of genetic marker on which directed studies had been conducted.

#### Combining scores

As direct measurements for comparing genetic variation in susceptibility across diseases were not available, we employed ordinal scales to estimate relative magnitudes of genetic variation. We ranked pedigree-based heritability estimates on the following five-point scale: zero (non-significant), low (less than 10 percent), intermediate (10 to 25 percent), high (25 to 50 percent), or very high (greater than 50 percent). We ranked marker-based odds ratios on a three-point ordinal scale: zero (non-significant), low (less than two), or high (greater than two). The overall scores for variation in susceptibility were based on the mean of the assigned scores for pedigree- and marker-based studies. Where only one type of evidence was available, estimates were based on only one score.

#### Certainty scores

Because of the caveats mentioned above, estimates for pedigree- and marker-based studies were additionally assigned “certainty scores” based on the relative reliability of the data reflected by the context and thoroughness of the study being used for the estimate [44]. Certainty scores were based on a five-point ordinal scale, with one indicating weak evidence from poorly-conducted studies and five indicating well-quantified data supported by multiple independent investigations. These certainty scores were included as weights in the estimates of genetic variation so that the overall estimates were weighted arithmetic means of the scores for each independent line of evidence for which data were available. We first calculated a weighted average of the marker-based data using the estimates and certainty scores for the four classes of genetic markers. We then calculated the overall estimate as a weighted mean of the heritability score and the marker-based average, each weighted by the corresponding certainty score (the weight for the marker-based data was the average of the weights for the 4 component classes of markers).

### Estimation of disease traits

The data on disease characteristics were compiled from online databases related to disease (CDC, WHO, GIDEON), texts of human infectious diseases [48]–[54], and, when necessary, primary literature on particular diseases. Whenever possible, estimates were based on data from the pre-antibiotic, pre-vaccine era to best reflect long-term evolutionary conditions. We collected data on traits relating to the following (see Information S1 for details).

### Disease effects on host fitness

- Mortality. Disease-induced mortality was estimated from case fatality rates for untreated infections. Where case fatality rates were unavailable, mortality was estimated from the annual death rate, expressed as a percentage of the number of annual cases.
- Fertility. Disease-induced male or female infertility and offspring mortality were estimated on a four point scale (see Information S1) based on clinical manifestations of the disease.

### Epidemiological and historical scope

- Lifetime risk of infection. The lifetime risk of acquiring a disease was estimated from sero-prevalence, prevalence, and incidence data.
- Geographical range. The geographical range of a disease was estimated as the number of countries where the disease is potentially endemic, as reported by GIDEON.
- Historical record of disease. The years since the disease was first recorded in human populations was estimated from historical reports reviewed by Kiple [51].

### The type of immune response evoked by infection

- Incubation period. This was estimated as the number of days from initial infection to onset of disease symptoms.
- Infectious period. The length of the infectious period was estimated as the average number of days for which infected individuals were capable of transmitting disease.
- Likelihood of re-infection. The lifetime risk of becoming re-infected with a disease after initial infection was estimated based on reports of re-infection rates.
- Vaccine status. Progress towards developing an effective vaccine against a disease was estimated according to the following scoring system: 1. Attempted, but no human trials; 2. Human clinical trials underway; 3. Partially-effective/strain-specific vaccine in use; 4. Effective vaccine in use.

#### Infectious agent classification

Etiological agents of each disease were categorized as RNA viruses, DNA viruses, bacteria, unicellular eukaryotes (protozoa), or multicellular eukaryotes (fungi and helminths). Infectious agent classification was treated as a class variable in the analyses.

#### Pathogen transmission mode

The following transmission modes were distinguished: sexual contact, close contact (including transmission via air or droplet), indirect contact (including fecal-oral or environmental transmission), and vector transmission. Transmission mode was treated as a class variable in all analyses, and no rankings were assigned to the classes.

### Validation

The results of the data validation are presented in the Information S1.

#### Susceptibility

We used three recent textbooks that reviewed evidence of variation in susceptibility to infectious diseases [8], [13], [16], and compared our estimates of genetic variation to levels of variation documented in the textbooks by totaling the proportion of pages in the texts devoted to variation in susceptibility to a given disease. We compared the relationship between our scores of variation in susceptibility and our estimates of the disease traits to independent estimates from previous work using Spearman's rank correlations.

#### Study Effort

We tested the relationship between the scores for variation in susceptibility and study effort for each disease by calculating Spearman's Rank Correlation for the scores and the number of citations listed in the PubMed database for each disease. The number of citations for a disease included all references returned in a PubMed search (as of July 2010) using the disease name as the search criterion.

### Analysis

The individual effects of the disease characteristics on variation in susceptibility were tested using Spearman's Rank Correlation for each of the continuous or ordinal disease traits. Effects of the class variables (infectious agent classification and pathogen transmission mode) were evaluated by analysis of variance (ANOVA), using the Tukey-Kramer correction for multiple comparisons to identify classes contributing to any observed differences. We conducted PCA to investigate the relative importance of the disease traits in determining the amount of variation in susceptibility (see Results Section for details).

The explanatory variables in the data set were significantly non-normal. We performed a log transformation of the data on the incubation period, infectious period, and mortality variables, which greatly improved the distributions but failed to fully restore normality to the data. Furthermore, we were unable to identify transformations that normalized the remaining variables in the data set, and therefore untransformed data was used; therefore, significance levels should be interpreted conservatively.

Unless otherwise noted, all data analyses were conducted using SAS version 9.2 [55] and R [56].

## Supporting Information

Information S1 Methodological approaches: validation, explanation, and discussion.
(DOC)
