# Supplementary material for: Evolutionary Determinants of Genetic Variation in Susceptibility to Infectious Diseases in Humans
Source: PLoS One. 2012 Jan 5;7(1):e29089. doi: 10.1371/journal.pone.0029089 (PMC3252296; doi:10.1371/journal.pone.0029089)
Supplement: Information S1 — Methodological approaches: validation, explanation, and discussion. (DOC) [file pone.0029089.s001.doc]

**S1. Methodological approaches: validation, explanation, and discussion**

**Supplement to**

Evolutionary determinants of genetic variation in susceptibility to infectious disease in humans

**Authors**

Christi Baker and Janis Antonovics

**INDEX**

1. Validation of Estimates

a. Levels of variation in susceptibility

b. Disease characteristics

c. Study effort

2. Explanation and Caveats for Measures of Genetic Variation in Susceptibility

a. Pedigree based studies

b. Marker based studies

c. Explanation of disease traits

3. Discussion of the Methodological Approach

**1. Validation of Estimates**

**a. Levels of variation in susceptibility**

We compared the relationship between our scores of variation in susceptibility and independent estimates from two types of previous studies. (a) We used three recent textbooks that reviewed evidence of variation in susceptibility to infectious diseases [1-3], and compared our estimates of genetic variation to levels of variation documented in the textbooks by totaling the proportion of pages in these texts devoted to variation in susceptibility to a given disease. There was a significant relationship between our scores and these independent assessments (Spearman's Rank Correlation: *r_s_* =0.34, *p*=0.04). (b) We compared our data to data previously (but independently) collected as part of an upper-level class project by undergraduate students at the University of Virginia (J. Antonovics, unpublished data). The methods employed for evaluating the literature and assigning the scores were different in the two studies, and therefore the absolute values assigned to each disease are not comparable; however, the relative estimates were strongly correlated (Fig. 1S).

**Fig. 1S.** Relationship between scores for variation in disease susceptibility estimated by two independent assessments (*r_s_*=0.47, *p*=0.009). On the x-axis are scores estimated using the methods described. On the y-axis are scores independently estimated as part of a upper-level class of project prior to the present study.


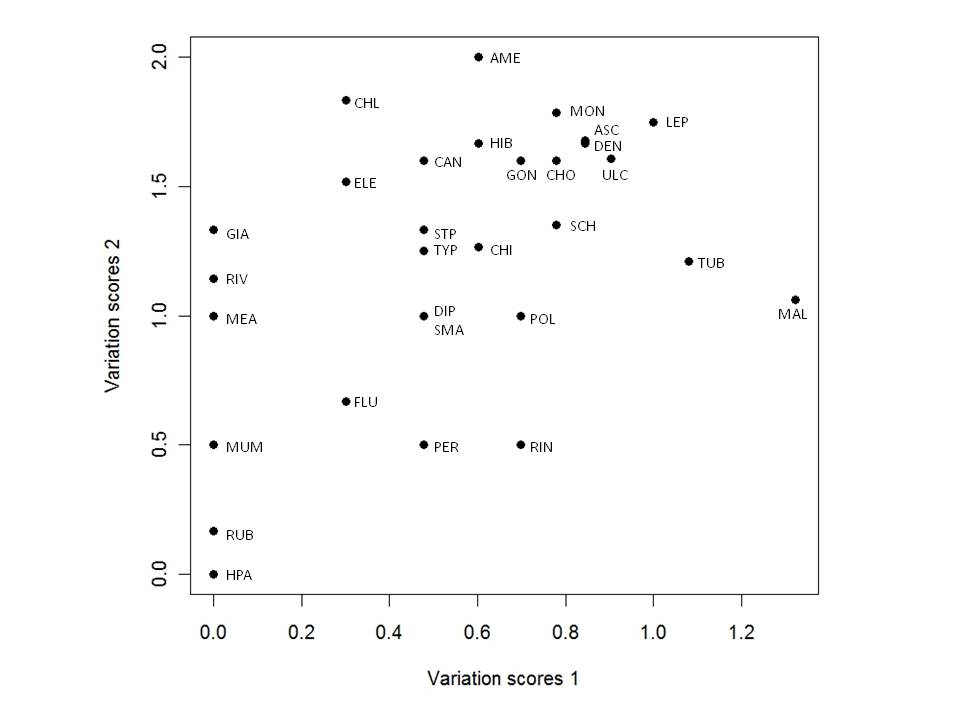


**b. Disease characteristics**

We also considered the reliability of our estimates of the disease characteristics by comparing our data to estimates of the same traits collected independently as part of the class project at the University of Virginia. Five disease traits were common to both data sets, and the independent estimates were significantly correlated for each of the five traits, including duration of infectious period (*r_s_* =0.80, *p*<0.001), effectiveness of vaccine development (*r_s_* =0.84, *p*<0.001), likelihood of re-infection (*r_s_* =0.73, *p*<0.001), mortality and reproductive effects (estimates were summed in order to be comparable to the class estimates, which combined these host fitness effects, *r_s_* =0.54, *p*=0.002), and geographical range (*r_s_* =0.44, *p*=0.01).

**c. Study effort**

We examined the relationship between our scores for variation in susceptibility and the amount of literature available for each disease in order to ensure that the variation in the scores was not simply due to differences in study effort among diseases. We found no correlation between the scores and study effort, which we measured as the number of citations returned in a PubMed search of each disease (*r_s_*=-0.17, *p*=0.29; Figure 2S). Therefore, we did not control for study effort in the analyses.

**Fig. 2S.** Relationship between study effort and scores for genetic variation in disease susceptibility (*r_s_*=-0.17, *p*=0.29). Study effort for a given disease was measured as the number of citations returned in July 2010 by the PubMed database search using the disease name as the search criterion.


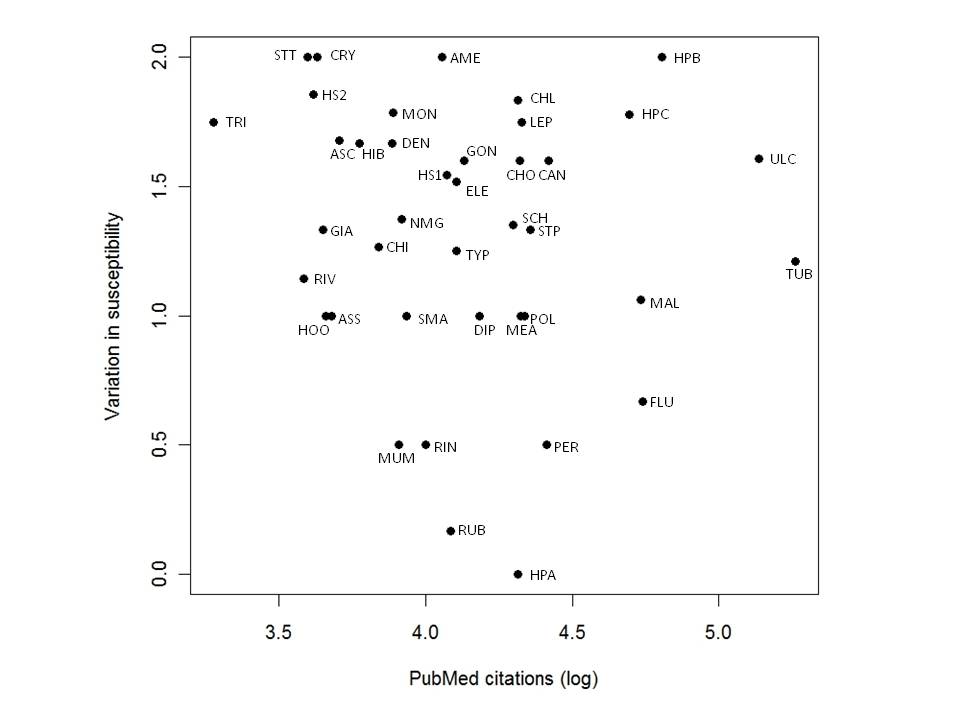


**2. Explanation and Caveats for Measures of Genetic Variation in Susceptibility**

**a. Pedigree-based studies**

Pedigree-based studies often estimated heritability directly, reporting a relative measure of total genetic variation in disease susceptibility for the study population.

Alternatively, many twin studies reported qualitative findings of a genetic component to susceptibility based on higher concordance in mono- versus dizygotic twins, but also presented the raw data necessary for more rigorous calculation of heritability estimates. In several cases, heritability could be approximated based on mono- and dizygotic twins’ probandwise concordance rates, defined as the proportion of co-twins infected given independent ascertainment of all twin individuals [4, 5]. To obtain unbiased data sets, this method requires that all twin pairs in the study population must be identified and independently ascertained, although these assumptions are often violated due to the limited availability of large-scale twin registries that would allow for complete ascertainment. Furthermore, heritability estimates based on probandwise concordance rates require knowledge of the number of healthy-healthy concordant twin pairs. Because of the difficulties in identifying all twin pairs in a population, many studies identified infected individuals and then ascertained data on twin status, thereby biasing the data set and making it impossible to estimate heritability. A further caveat to twin study estimates of heritability is that these estimates are likely to be inflated by environmental variance, as they only account for variance in the common environment shared within families and do not account for variance among families. Furthermore, heritability estimates are dependent on uniform disease risk; however, disease exposure is generally unknown, particularly for healthy-healthy concordant twin pairs.

**b. Marker-based studies.**

Marker-based studies typically reported odds ratios, which reflect variation in disease risk associated with the particular genetic markers. Variation in disease risk associated with a single marker locus is evidence that there are genetic contributions to overall genetic variation in susceptibility, but estimating the magnitude of this contribution is difficult. Because heritability estimates are a function of genetic effect sizes and allele frequencies, genetic markers are most likely to be important contributors to total genetic variation if gene effects are large, markers are in strong linkage disequilibrium with the susceptibility alleles, alleles are sufficiently common, and disease risk is high. However, translating odds ratios into quantitative estimates of the contribution of a particular marker to overall genetic variation is fraught with difficulties. Heritability can be estimated using marker-based data by assuming that disease status is a threshold character determined by underlying continuous variation in [6]. Under this theory, heritability can be estimated for a marker locus based on differences in disease incidence among groups carrying different alleles. However, calculating genetic variation requires knowledge of allele frequencies, and this was often unknown because the goal of case-control studies was usually allele identification for further molecular characterization. These studies systematically recruited designated numbers of infected and healthy individuals and usually did not report overall population allele frequencies, making it impossible to estimate heritability for the marker loci reported. Furthermore, valid estimates of the contribution of these markers to genetic variance necessitates that all individuals in a study group experience equal pathogen exposure, as allelic effect sizes will likely be underestimated if susceptibility alleles are over-represented in healthy control groups due to inclusion of unexposed individuals. Heritability estimates based on these data come with the further caveat that they are relevant only within the fraction of the population at risk for the disease. These estimates can be adjusted to account for disease risk in the population based on prevalence data, though these data are rarely reported in the literature. Further complications arise in evaluating variation identified at multiple loci, because rigorous assessment of their net contribution to heritability requires additional information about whether their effects are additive or if there are epistatic interactions, as well as information on their linkage disequilibrium.

The many caveats associated with the various data sources make it very difficult to rationally integrate pedigree-based studies and marker-based studies into a single estimate for each disease. Heritability estimates and studies of genetic variation in disease susceptibility must be interpreted with caution, as they are dependent on environmental variance, including variation in pathogen exposure. Furthermore, association studies are often difficult to replicate due to genetic differences among populations, uncertainty about linkage between the markers and genes determining susceptibility, and ambiguity concerning epistatic effects [7]. However, substantial heritability estimates, particularly when supported by molecular evidence for genetic variants associated with disease, provides strong evidence for genetic variation in susceptibility. Hence, the overall scores for variation in susceptibility were based on the mean of the assigned scores for pedigree- and marker-based studies. Where only one type of evidence was available, estimates were based on only one score.

**c. Explanation of disease traits**

***Mortality*.** Disease-induced mortality was estimated from case fatality rates for untreated infections. Where case fatality rates were unavailable, mortality was estimated from the annual death rate, expressed as a percentage of the number of annual cases. Benign diseases (e.g. candidiasis, ringworm, leprosy, herpes simplex viruses 1 and 2) were never associated with quantitative mortality data and qualitative reports indicated only that fatalities were extremely rare. These diseases were assigned fatality rates of one in a million cases in order to rank them below all other diseases for which mortality data was available.

***Fertility.*** Disease-induced fitness effects due to male or female infertility and offspring mortality were almost never reported quantitatively, and therefore they were estimated using the following scoring system based on reports of the clinical manifestations of the disease: 1. Effects unreported in clinical manifestations or studies have found no effect; 2. Effects are mild/rare and short-lived; 3. Effects are extreme/common but short-lived; 4. Effects are mild/rare and long-lasting; 5. Effects are extreme/common and long-lasting. ***Lifetime risk of infection.*** The lifetime risk of acquiring a disease was estimated from seroprevalence, prevalence, and incidence data. Seroprevalence reports were the preferred source of information, as these figures captured currently infected individuals as well as those with a history of disease. For some diseases, particularly diseases with long infectious periods, seroprevalence figures were not available. In these cases, prevalence or incidence data were used. For long-duration infections, prevalence is likely to give a reasonable approximation of lifetime risk since infected individuals recover slowly and are retained in prevalence estimates for a long period of time. Where incidence data were the only sources of information, estimates of lifetime risk of infection were made by calculating the annual cases reported worldwide for a disease as a fraction of the global population size in the given year.

***Geographical range.*** The geographical range of a disease was estimated as the number of countries where the disease is potentially endemic, as reported by GIDEON. No correction was made for the population size or for the land-area of the individual countries.

***Historical record of disease.***  The years since the disease was first recorded in human populations was estimated from historical reports reviewed by Kiple [8]. For diseases with distinct symptoms, these records were fairly straightforward. However, many diseases were difficult to distinguish in historical records due to similarity in the symptoms (e.g. diarrheal diseases can have multiple causes). Although phylogenetic analyses might have reconciled some of these uncertainties, we did not consider estimates of disease age based on these molecular data because they were not consistently available for all of the diseases included in the present study.

***Incubation period.*** This was estimated as the number of days from initial infection to onset of disease symptoms. Incubation period was used because latent period (the time from infection to actual disease transmission) was seldom reported in disease texts. For soil-transmitted helminthic diseases, estimates were based on reports of the pre-patent period (time from infection to egg production by adult helminth) which were consistently reported with little variability.

***Infectious period.*** The length of the infectious period was estimated as the average number of days for which infected individuals were capable of transmitting disease. Where infectious period was not reported, estimates were made based on the duration of disease symptoms and qualitative reports of infectious period relative to duration of symptoms.

***Likelihood of re-infection.*** The lifetime risk of becoming re-infected with a disease at some time after initial infection was estimated using the scoring system below. Where available, reports of re-infection rates were used to obtain quantitative estimates of the percent of individuals that experience subsequent infection after an initial disease episode. Where percentages were not available, ranks were assigned based on qualitative descriptions of the likelihood of re-infection or the duration of post-infection immunity. The following scale was used to estimate the occurrence of reinfection: 1. Never/rare (0-1%); 2. Occasional (1-10%); 3. Common (10-50%); 4. Usually (50-100%).

***Vaccine development.*** Progress towards developing an effective vaccine against a disease was estimated according to the following scoring system: 1. Attempted, but no human trials; 2. Human clinical trials underway; 3. Partially-effective/strain-specific vaccine in use; 4. Effective vaccine in use. Ranks were assigned in one-half increments to allow for intermediate scenarios (e.g. human trials for vaccines reported to be only partially effective were assigned a rank of 1.5).

***Infectious agent classification.*** Etiological agents of each disease were categorized as RNA viruses, DNA viruses, bacteria, unicellular eukaryotes (protozoa), or multicellular eukaryotes (fungi and helminths). The classes were ranked in this order based on differences in average mutation rate per base pair [9, 10], which were used as surrogates for differences in the site-specific amount of genetic variation among pathogen types. This classification was treated as an ordinal variable based on decreasing levels of mutation associated with the rank order, but infectious agents were also considered as a class variable to examine the possible effects of different pathogen types unrelated to the associated levels of pathogen genetic diversity.

***Pathogen transmission mode.*** The following transmission modes were distinguished: sexual contact, close contact (including transmission via air or droplet), indirect contact (including fecal-oral or environmental transmission), and vector transmission. Transmission mode was treated as a class variable in all analyses, and no rankings were assigned to the classes since we had no a priori assumptions about underlying mechanisms that might generate differences in levels of genetic variation in susceptibility to disease of different transmission modes**.**

**3. Discussion of the Methodological Approach**

Precise quantification of the differences in levels of variation for susceptibility was difficult. The relevant genetic data were generally collected for other purposes and were not always amenable to comparison across studies or across diseases. Reports often omitted information, such as allele frequencies and disease prevalence in the general population, which are vital for estimating contributions of genetic effects in susceptibility to overall levels of variation. Quantitative estimates of overall levels of variation were available for a few diseases, but even then estimating heritability of susceptibility to infectious diseases was complicated by a lack of knowledge about levels of disease exposure or the impact of environmental differences that might be manifested as familial effects. While the technical and statistical sophistication of modern investigations was often impressive, the mitigating effects caused by contemporary vaccination regimes and other medical interventions made evolutionary interpretations more difficult.

We carried out as rational a search as possible using different types of evidence for variation in susceptibility. Every attempt was made to be clear and objective in evaluating and weighing the various data sources and different methodologies. However, perfection was impossible, and many of the assumptions made in the assessments may require further consideration as additional information becomes available. For instance, we could not account for epistatic effects, although there are likely to be genetic interactions among susceptibility loci. For example, the sickle cell trait and α-thalassaemia each confer protection against malaria when inherited alone but offer no protective effect when inherited together [11].

In spite of the caveats, comparisons of the scores to various independent evaluations of variation in susceptibility indicate that the estimates are accurate at least with regard to *relative* levels of variation across multiple diseases.

The disease characteristics were less problematic to estimate, but surprisingly it was difficult to find consistent quantitative estimates across all the diseases. Amid the vast quantities of medical literature relevant to physicians and patients, we were faced with a paucity of “evolutionarily-relevant” data. We found it difficult to obtain good estimates for the mortality caused by a number of diseases, such as dengue fever because mortality rates were reported only for hospitalized patients representing severe cases, and difficult to determine the sterility effects of, for example, gonorrhea because comprehensive follow-up studies on patients had not been conducted. Some of the issues were more intrinsic to the transient and variable nature of disease incidence. Epidemiological characteristics related to prevalence and risk of infection are highly variable, both temporally and geographically. New research efforts such as the WHO Global Burden of Disease analysis are aimed at quantifying disease characteristics associated with human diseases [12, 13], but these estimates are generally too contemporary to reflect pre-vaccine and pre-antibiotic conditions, and the data are not always attributable to specific pathogen species.

The approach we used to estimate the disease characteristics followed that of Lockhart et al. [14], converting raw data into numerical ranked values that allowed the incorporation of qualitative data from reports that included those for which quantitative data were not consistently available. We collected data from multiple independent sources and performed additional literature searches to resolve discrepancies between these sources. In this way, we obtained data on the traits of interest for nearly all diseases, with the exception of data on reproductive effects, for which the trait estimates were often inferred merely by omission.

**References**

1. Kimman TG (2001) Genetics of infectious disease susceptibility. Boston: Kluwer Academic Publishers. 247 p.

2. Kaslow RA, McNicholl J, Hill AVS (2008) Genetic susceptibility to infectious diseases. New York: Oxford University Press. 447 p.

3. Bellamy R (2004) Susceptibility to infectious diseases: The importance of host genetics. Cambridge: Cambridge University Press. 398 p.

4. McGue M (1992) When assessing twin concordance, use the probandwise not the pairwise rate. Schizophr Bull 18: 176.

5. Smith C (1974) Concordance in twins: Methods and interpretation. Am J Hum Genet 26: 454-466.

6. Falconer DS, Mackay TFC (1996) Introduction to quantitative genetics. Essex: Longman. 464 p.

7. Greene CS, Penrod NM, Williams SM, Moore JH (2009) Failure to replicate a genetic association may provide important clues about genetic architecture. PLoS One 4: e5639.

8. Kiple KF (1993) The Cambridge world history of human disease. Cambridge: Cambridge University Press. 1176 p.

9. Drake JW (1991) A constant rate of spontaneous mutation in DNA-based microbes. Proc Natl Acad Sci USA 88: 7160-7164.

10. Gago S, Elena SF, Flores R, Sanjuan R (2009) Extremely high mutation rate of a hammerhead viroid. Science 323: 1308.

11. Williams TN, Mwangi TW, Wambua S, Peto TE, Weatherall DJ, et al. (2005) Negative epistasis between the malaria-protective effects of alpha+-thalassemia and the sickle cell trait. Nat Genet 37: 1253-1257.

12. Lopez AD, Mathers CD, Ezzati M, Jamison DT, Murray CJL (2006) Global burden of disease and risk factors. New York: Oxford University Press. 475 p.

13. Mathers C, Fat DM, Boerma JT, World Health Organization, ebrary I (2008) The global burden of disease. Geneva: World Health Organization. 146 p.

14. Lockhart AB, Thrall PH, Antonovics J (1996) Sexually transmitted diseases in animals: Ecological and evolutionary implications. Biol Rev Camb Philos Soc 71: 415-471.
